# Regulating the Expansion Characteristics of Cementitious Materials Using Blended MgO-Type Expansive Agent

**DOI:** 10.3390/ma12060976

**Published:** 2019-03-25

**Authors:** Peng Liu, Zhiyang Chen, Min Deng

**Affiliations:** College of Materials Science and Engineering, Nanjing Tech University, Nanjing 210009, China; pengl@njtech.edu.cn (P.L.); zychen@njtech.edu.cn (Z.C.)

**Keywords:** MgO, compacted body, expansion stress, blended MgO

## Abstract

To promote the application of MgO-type expansive agents (MEAs), the expansion stresses produced by compacted MEAs with different activities cured in water at 40 °C were measured using a self-designed expansion stress test apparatus. Based on these, different MEAs were divided into the early-type MgO expansive agent and the late-type MgO expansive agent classifications according to the stress curves of compacted MEAs. The two types of MEAs were blended with each other at different ratios and added into cement pastes. Results indicated that the expansion of the cement pastes added with blended MEAs lasted from the beginning to 200 days later, and the expansion characteristics can be regulated by adjusting the blending ratio of MEAs and the choice of types of MEAs. The results suggest that the expansion of MEAs can be improved by using blended MEAs in practical applications.

## 1. Introduction

Decades of research have produced success in the application of MgO-type expansive agents (MEAs). In China, MEAs have been applied in different parts of more than 60 dams and even into some entire dams [1,2,3], due to the chemically stable hydration products, relatively low water requirement for the hydration of MEAs, designable expansion property, and no obvious shrink in the late age [4,5,6].

Engineers have sought an expansive agent that can generate a required expansion to compensate for the specific shrinkage of concrete. MEAs outperform other expansion agents especially in terms of the designable expansion property. Therefore, MEAs can be a good choice for construction projects. However, current design measures are simple and relatively ineffective. One common method is to change the calcining temperature of magnesite [4,7,8], since MEAs prepared at different calcining temperatures exhibit different expansion characteristics in concrete. However, it is difficult to meet the needs of some special projects and the effect of this method is not obvious. Some high-activity MEAs calcined under lower temperatures can compensate for the concrete shrinkage in the early age, but the effect is not obvious in the late age. In comparison, low-activity MEAs calcined under higher temperatures do not work in the early age; they can compensate for the concrete shrinkage in the late age, but the soundness of concrete is a potential problem due to the huge expansion in the late age [9,10]. Thus, the effect of changing the calcination temperature is not obvious. Modification is another method to regulate the expansion property of MEAs. Wu et al. [11] modified MEAs with a C-18 unsaturated fatty acid; this type of MEA can only hinder the early hydration of the MEA and reduce the amount of early expansion. Ye et al. [12] prepared cement pastes with nano-MgO, and found that when the content of nano-MgO reached 8%, the soundness of concrete was still acceptable. Pei et al. [13] proposed a mixture of paraffin and MgO, showing the admixtures can significantly compensate for the early shrinkage and reduce thermal accumulation. However, modification is too expensive, which will restrict the use in practical applications.

CaO-based expansive agents and sulfoaluminate-type expansive agents can also be effectively used to compensate for the shrinkage of concrete, but they can only produce expansion during the early age [14]. Some MEAs with low activity can produce slower expansion, so Mo et al. [15] prepared blended expansive agents with MgO and CaO. They found that the cement paste can produce rapid expansion and the expansion can be regulated by changing the blending ratio of MgO and CaO. Xu et al. [16] prepared an expansive agent that contains MgO, CaO and C_2_S, but the amount of CaO was low, so the regulation of expansion characteristics of cementitious materials was not obvious. So, blending different types of expansive agents is a method to regulate the expansion of cementitious materials, but the differences in expansion mechanisms may complicate the control of the expansion. Therefore, in this paper, a novel method is proposed that involves blending different MEAs with different activities to regulate the expansion characteristics of cementitious materials. Because the expansion property of an MEA in practical applications is dependent on the activity, it is possible to select and blend MEAs with different activities to achieve the desired expansion characteristics in the cementitious materials.

When selecting MEAs to blend, we must have guidance for the MEA selection. When applied in concrete, a low-activity MEA expands less at the early age, but expands heavily at the late age, whereas a high-activity MEA shows the opposite expansion property. However, the expansion characteristics of cementitious materials with MEAs are also affected by cement shrinkage, the alkali–aggregate reaction, and the physical expansion, so we do not know how much of the expansion characteristics are contributed by the MEA. Therefore, studying the expansion property of MEAs is necessary to understand the expansion development of MEAs with different activities at different ages.

Proper types of MEAs for blending should be selected according to the expansion property. In this study, an expansion stress test apparatus was designed to test the stress development of MEAs. Then, the MEAs were classified into different types for selection for blending, which can compensate for both the early shrinkage and late shrinkage of concrete. The expansion stress of a compacted MEA provides a novel method to characterize the expansion property of the MEA, because the existing characterizations of the MEA expansion property focus on the activity of the MEA and the content of periclase in the MEA, so do not fully reflect the expansion property of the MEA [17,18,19,20]. Therefore, the means of characterization need to be improved and should be more related to the expansion property of the MEA, so the MEA is easier to use in some projects.

## 2. Materials and Methods

### 2.1. Materials

Magnesite from Liaoning Province, China was selected as the raw material, and its chemical compositions are shown in Table 1. Magnesite was calcined at different temperatures to prepare four types of MEAs, named as MEA-71, MEA-101, MEA-375 and MEA-1250. The chemical compositions of the MEAs are shown in Table 1 and the physiochemical properties are shown in Table 2. The periclase contents of MEAs were analyzed by Rietveld quantitative X-ray diffraction (XRD; 40 kV, 30 mA, Cu-Kα radiation). The specific surface area of the MEA was tested by fully auto surface area analyzer (BET, Micromeritics, Norcross, GA, USA). The average crystallite size of the MEA was tested by XRD (Thermo Electron Corporation, Waltham, MA, USA). The activity of the MEA was determined as the neutralization time of the MEA to completely react with citric acid. Type P.II 52.5 Portland cement produced by Jiangsu-Onada Cement Corp. (Nanjing, China) was used here and the chemical composition is shown in Table 1.

### 2.2. Apparatus

#### 2.2.1. Expansion Stress Test Apparatus

**Figure 1:**
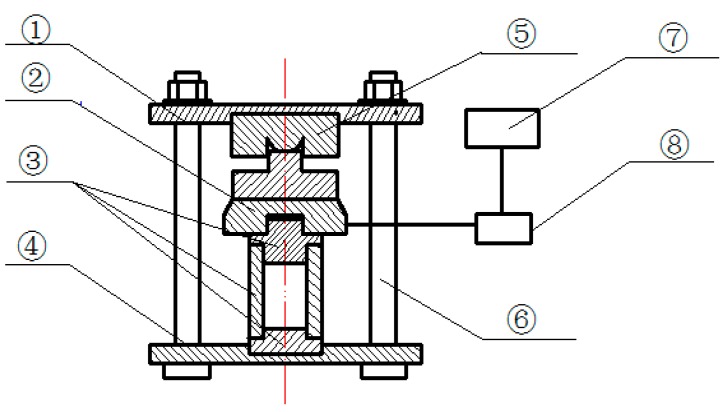
Schematic diagram of the expansion stress test apparatus: ① top plate, ② sensor, ③ sample mold, ④ bottom plate, ⑤ anti-load measuring head, ⑥ constrained screw, ⑦ data acquisition system, and ⑧ transmitter.

The expansion stress test apparatus includes a sensor, a transmitter, a data acquisition system, an anti-load measuring head, and a sample mold. The schematic diagram of the tester is shown in Figure 1.

**Figure 2:**
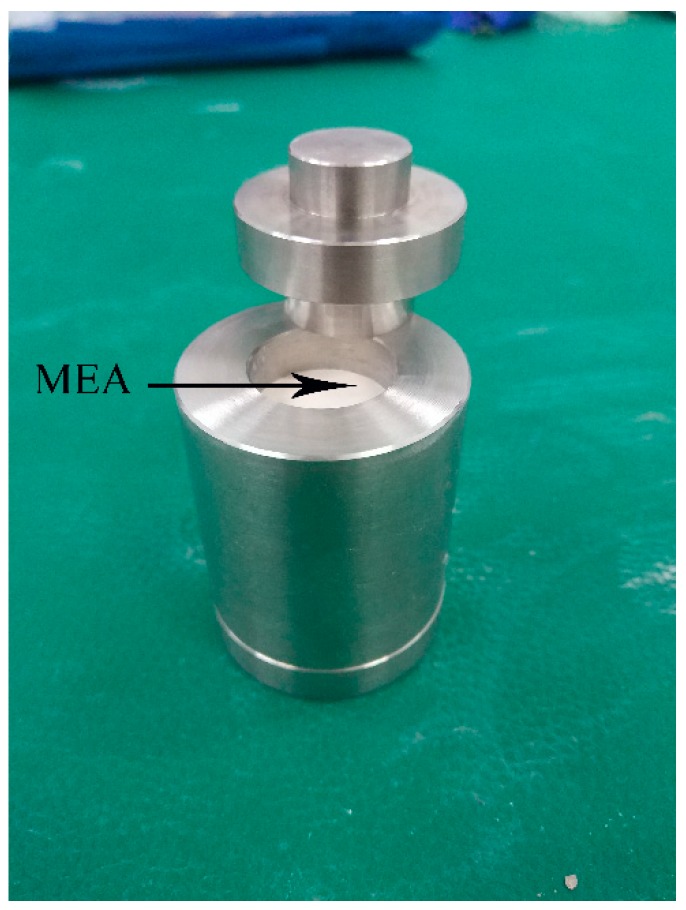
Physical sample mold.

The sample mold to measure the expansion stress of the MEA was made of CrWMn steel with hardness of HRC61 and elastic modulus of 224 GPa (Figure 2).

#### 2.2.2. Expansion Stress Testing

Forty grams of each type of MEA was put into the sample mold, which was compacted under a pressure of 464 MPa by a press, which was maintained for 5 s. The thicknesses of compacted MEA-71, MEA-101, MEA-375 and MEA-1250 were 34.1, 31.7, 28.2 and 25.4 mm, respectively. Then, the mold was placed into the expansion stress apparatus, and a pressure of 2 ± 0.1 MPa was applied by tightening the nut to pre-tighten the tester. Then, the apparatus was cured in a constant temperature curing box in water at 40 °C. The expansion stress (*σ*, MPa) was calculated according to Equation (1):

(1) $$\sigma =\frac{{4(F}_{t}{\;-\;F}_{0})g}{\pi d^{2}},$$

where *F_t_* and *F*_0_ are the sensor values at time *t* and at the beginning, respectively (kg); *g* = 9.8 m/s^2^ is the gravity acceleration; *d* = (24 mm) is the inner diameter of the mold; and π = 3.14.

### 2.3. Microcosmic Analysis of Compacted MEA

To explore the hydration process of the four types of compacted MEAs, each MEA was prepared into a number of compacted MEAs: one was used to measure the expansion stress, the others were used to measure the hydration progress and the microstructure of compacted MEAs at certain curing age, and they were cured at the same situation. A sample was removed at a certain time and soaked in alcohol for 72 h to terminate the hydration of periclase in the compacted MEA. The compacted MEA was demolded by a press, then crushed and ground into fine particles less than 80 μm in size to investigate the hydration products by XRD. The hydration degree of periclase in the compacted MEA was calculated by thermogravimetric analysis and differential scanning calorimetry (TG–DSC) due to the content of brucite that was produced by the hydration of periclase.

The compacted MEAs were prepared as required to observe the pore size distribution by mercury intrusion porosimetry (MIP) at a certain age.

The microstructures of the fractured surface of compacted MEAs were examined by a JSM-5900 scanning electron microscope (SEM, JEOL, Ltd., Osaka, Japan).

### 2.4. Blending of MEAs

MEAs were classified into different types according to the expansion stress curves of compacted MEAs. Then, early-type MgO expansive agents (MEA-71 and MEA-101) and late-type MgO expansive agents (MEA-375 and MEA-1250) were mixed with each other at different ratios in a mixer for 24 h. A total of 12 types of blended MEAs were mixed and the blending ratios are outlined in Table 3.

### 2.5. Production of Cement Pastes

Four series of cement pastes (20 × 20 × 80 mm) according to Table 3 were produced and cured in water at 40 °C to explore the effects of expansion property with blended MEAs according to JC/T 313-2009 (Chinese standard) [21]. The total amount of blended MEAs by cement weight was 8% and the water/cement ratio is 0.3. The length changes of the cement pastes were measured at certain ages.

## 3. Results and Discussion

### 3.1. Expansion Stress of Compacted MEAs

**Figure 3:**
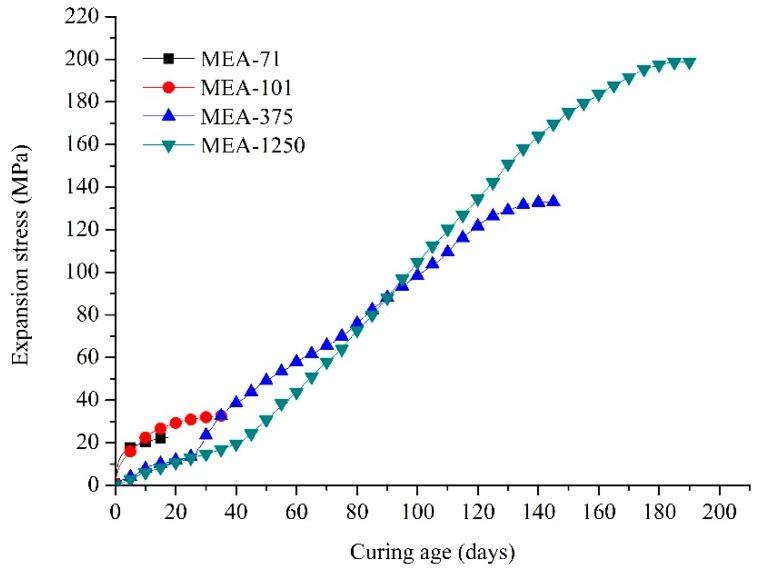
Expansion stress curves of compacted MEA-71, MEA-101, MEA-375 and MEA-1250 cured in water at 40 °C.

Figure 3 shows the development of the expansion stresses of compacted MEA-71, MEA-101, MEA-375 and MEA-1250 cured in water at 40 °C. The high-activity compacted MEAs (MEA-71 and MEA-101) generated expansion stress quickly in the beginning, but no significant expansion stress in the late age. The expansion stresses of compacted MEA-71 and compacted MEA-101 developed for about 18 and 34 days, respectively, and stabilized at 22.41 and 32.73 MPa, respectively. In contrast, for the low-activity compacted MEAs (MEA-375 and MEA-1250), the expansion stress developed very slowly and became significant only after many days. The expansion stress of compacted MEA-375 developed rapidly after 25 days, and that of compacted MEA-1250 needed about 40 days. The resulting stable expansion stress of compacted MEA-1250 was 198.74 MPa, which was greater than the 134.03 MPa of compacted MEA-375. The expansion stress of compacted MEA-1250 was maintained for 190 days, longer than the 147 days of compacted MEA-375.

For compacted MEA-71 and compacted MEA-101 with high activity, the expansion stress developed rapidly and stabilized for a short time, and the final stable expansion stress was small. Thus, they can be categorized as early-type MgO expansive agents that are suitable for compensating for the early shrinkage of cementitious materials. In contrast, for compacted MEA-375 and compacted MEA-1250 with low activity, the expansion stress developed slowly at an early age but faster at the late age, and stabilized for a long time. The final stable expansion stresses were larger, indicating they can compensate for the late shrinkage of cementitious materials. So, they were classified as late-type MgO expansive agents. The higher activity of compacted MEAs led to larger expansion stress in the early age but smaller expansion stress in the late age, and the expansion stress of MEAs developed for a shorter time for both the early-type MgO expansive agent and the late-type MgO expansive agent.

### 3.2. Hydration Process of Compacted MEAs

**Figure 4:**
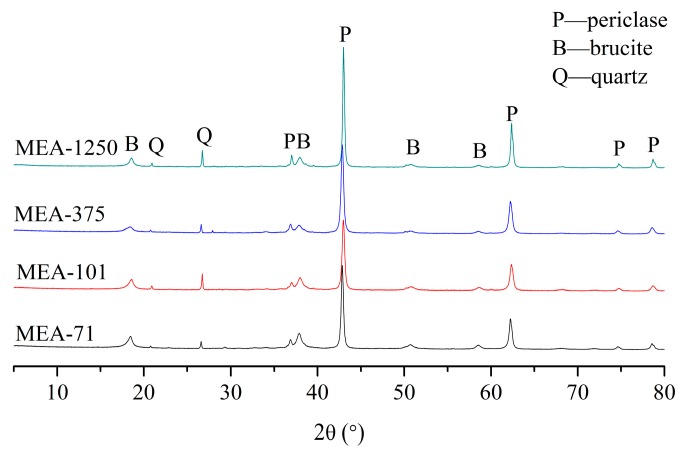
X-ray diffraction (XRD) patterns of compacted MEAs cured in water at 40 °C for 3 days.

**Figure 5:**
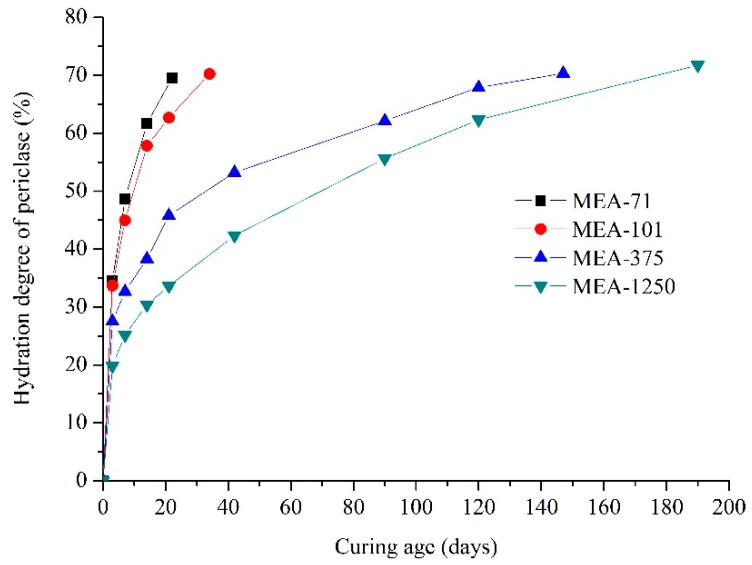
Hydration degree of periclase in compacted MEAs cured in water at 40 °C.

Figure 4 shows the XRD patterns of compacted MEAs cured in water at 40 °C for three days. Brucite existed in all four compacted MEAs, and its peak was more obvious in higher-activity MEAs. Figure 5 shows the hydration degree of periclase in compacted MEA cured in water at 40 °C. At three days, about 34.55% of the periclase in compacted MEA-71 and 33.69% in compacted MEA-101 were hydrated, but the corresponding hydration degrees of compacted MEA-375 and compacted MEA-1250 were 27.52% and 22.84% respectively. The expansion stress of compacted MEA-71 stabilized after 18 days and the hydration degree of periclase was 69.56%. The hydration degree of periclase in compacted MEA-101 reached 69.16% after 34 days. However, more days were needed when the expansion stress of compacted MEA-375 and compacted MEA-1250 tended to stabilize. The hydration degree of periclase in compacted MEA-375 was 69.34% at 147 days, and compacted MEA-1250 required 190 days to reach a hydration degree of periclase of 69.77%.

**Figure 6:**
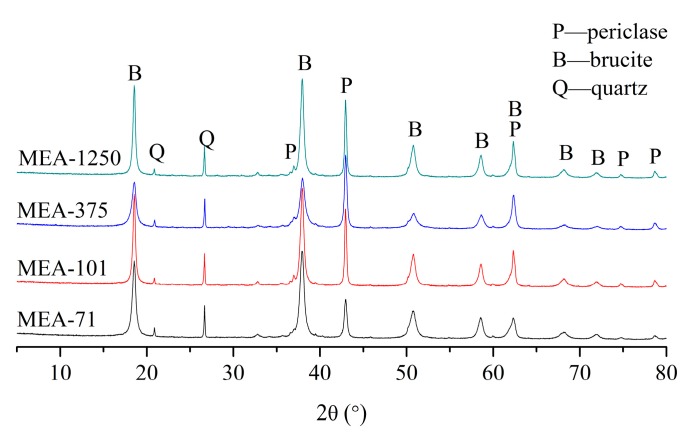
XRD patterns of compacted MEAs cured in water at 40 °C when the expansion stresses stabilized.

Generally, when the expansion stress of compacted MEA was stable, the hydration degree of periclase was about 70% for either the early-type MgO expansion agent or the late-type MgO expansion agent [17,19]. The XRD patterns of compacted MEAs when the expansion stress was stabilized are shown in Figure 6, and periclase was still present. The early-type MgO expansive agents (MEA-71 and MEA-101) hydrated quickly and the expansion stresses developed quickly. However, the late-type MgO expansive agents (MEA-375 and MEA-1250) hydrated slowly at first, and required a long time for the hydration degree to reach 70%. The hydration process of compacted MEAs is consistent with the development of the expansion stress of compacted MEAs.

### 3.3. Microstructure of Compacted MEAs

**Figure 7:**
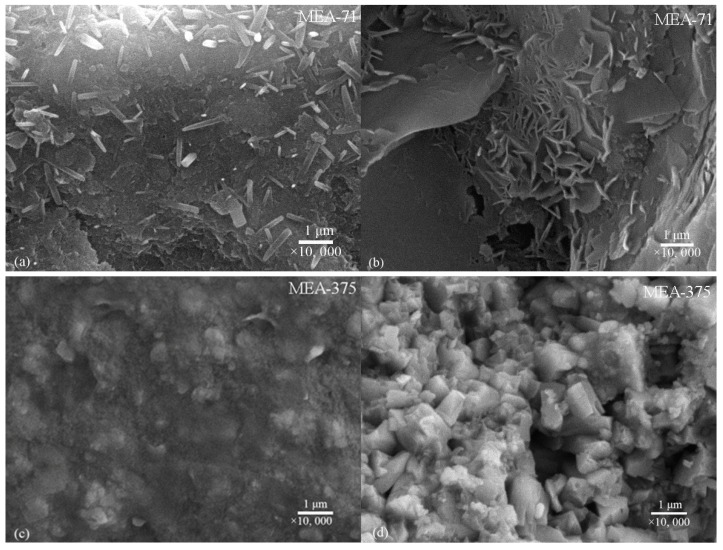
Scanning electron microscopy (SEM) images of the fracture surface of compacted MEAs cured in water at 40 °C when the expansion stresses stabilized: (**a**,**b**) compacted MEA-71, and (**c**,**d**) compacted MEA-375.

**Figure 8:**
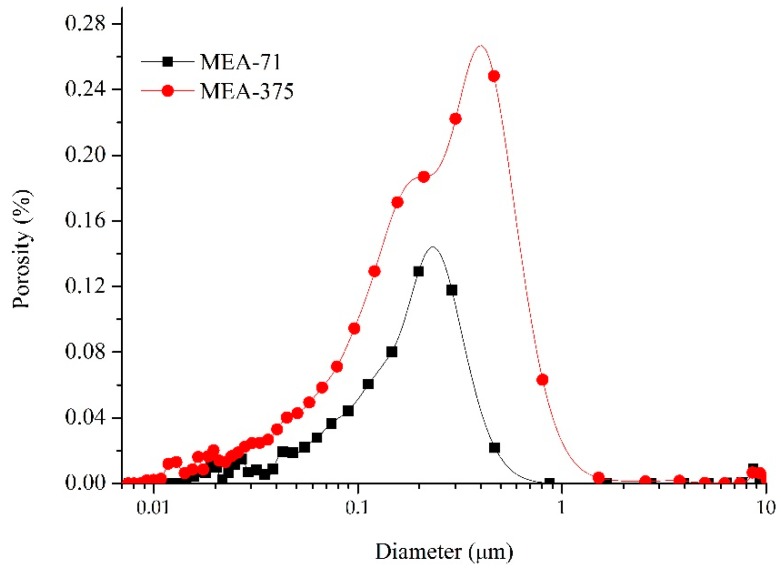
Pore size distribution of compacted MEA-71 and compacted MEA-375 cured in water at 40 °C when the expansion stresses stabilized.

Figure 7 shows the SEM images of the fracture surface of compacted MEA-71 (early-type MgO expansion agent) and compacted MEA-375 (late-type MgO expansion agent) cured in water at 40 °C when the expansion stresses were stabilized. The compacted MEAs all had very dense structures (Figure 7a,c). As the hydration proceeded, the periclase continuously hydrated into brucite and filled the pores, so the compacted MEAs became increasingly dense. The compacted MEA-71 and compacted MEA-375 were dominated by nanopores, and the total porosities were 5.81% and 5.03%, respectively (Figure 8), when the expansion stresses stabilized.

**Figure 9:**
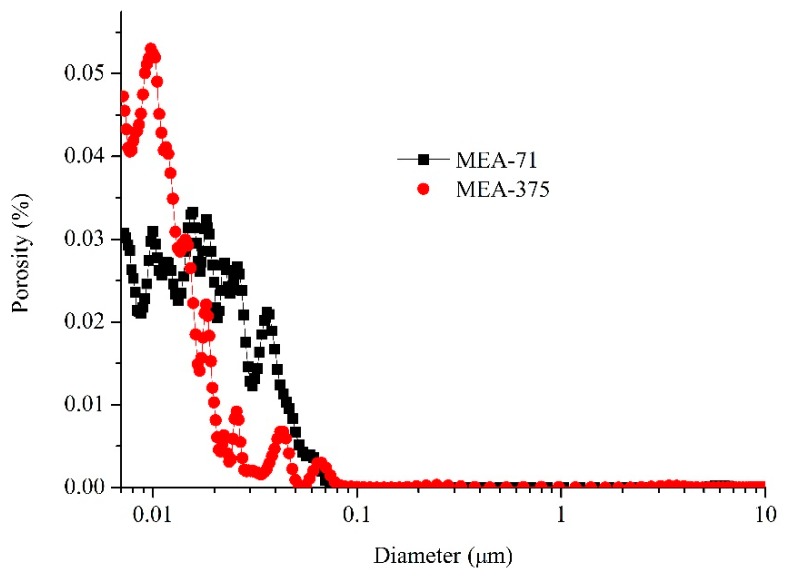
Pore size distribution of compacted MEA-71 and compacted MEA-375 cured in water at 40 °C for 3 days.

Despite the different activities, the compacted MEAs basically had the same dense structure, and at this point, the expansion stresses stabilized. The differences were the development process of expansion stresses, the brucite formation speed, and the morphology of the formed brucite. The early-type MgO expansive agent hydrated faster, the formed brucite quickly filled the pores of the compacted body, and a larger number of hexagonal plates of brucite generated together to form the dense structure. Figure 7b shows the brucite generated in the hole area where the hexagonal plates of brucite can grow free. The late-type MgO expansive agent hydrated slowly and cube-shaped brucite crystals formed in the hole area (Figure 7d). A dense structure was generated in most areas (Figure 7c). Figure 9 shows the pore size distributions of compacted MEA-71 and compacted MEA-375 cured in water at 40 °C for three days, and the total porosities were 17.21% and 40.20%, respectively, indicating that the compacted MEA-71 had much smaller pores than the compacted MEA-375 at an early curing age. However, when the expansion stresses of the compacted MEAs developed stably, the total porosities were almost the same. This indicates that the early expansion stress of the late-type MgO expansive agent developed slowly, and the expansion stress stabilized after a longer time.

### 3.4. Blending of MEAs

**Figure 10:**
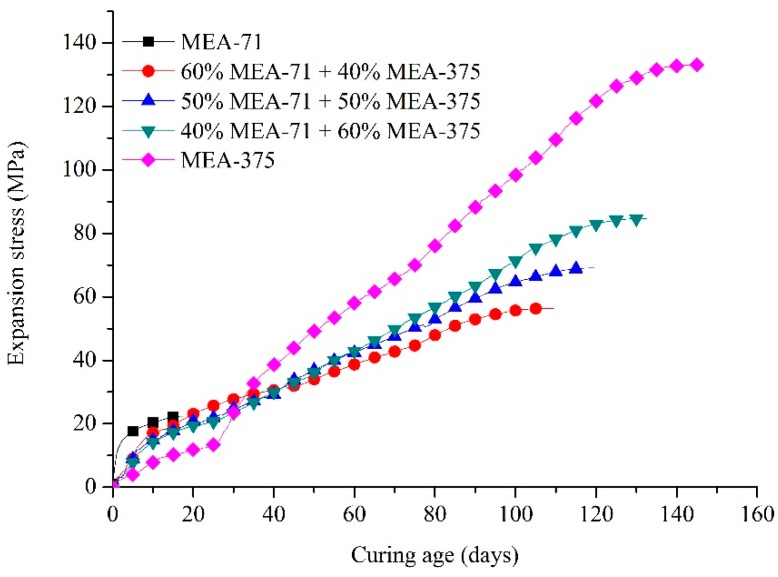
Expansion stress curves of blended compacted MEAs.

To study the change in expansion stresses of MEAs of different ages after blending, three types of blended compacted MEAs were tested for the development of expansion stresses. Figure 10 shows that before the age of 18 days, the expansion stresses generated by blended compacted MEAs were less than compacted MEA-71, but obviously greater than compacted MEA-375. After 34 days, they still produced expansion stresses, and did not produce huge late expansion stress, like compacted MEA-375. Thus, they exhibited expansion stresses that can develop from early to late ages. A higher proportion of MEA-71 in blended compacted MEA led to greater expansion in the early age, and lower expansion stress in the late age, and the expansion stress stabilized more quickly. So, the expansion stress of MEAs can be regulated by adjusting the blending ratio of the MEAs.

In summary, the expansion stresses caused by blended compacted MEAs were lower than the early-type MgO expansive agent (MEA-71) and greater than the late-type MgO expansive agent (MEA-375) before 18 days. After 34 days, the expansion stresses of blended compacted MEAs were all lower than compacted MEA-375. So, the effect of expansion stresses of compacted MEAs after blending were better than those without blending.

### 3.5. Expansion of Cement Pastes Containing Blended MEAs

**Figure 11:**
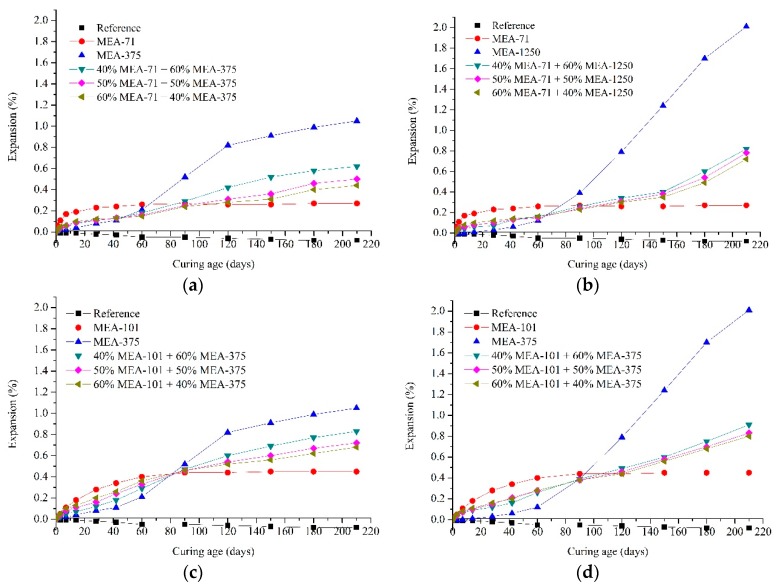
Expansion curves of cement pastes containing 8 wt % of blended MEAs cured in water at 40 °C: (**a**) MEA71 + MEA-375, (**b**) MEA-71 + MEA-1250, (**c**) MEA-101 + MEA-375, (**d**) MEA-101 + MEA-1250.

Figure 11 shows the certain regulation of expansion characteristics in cement pastes with blended MEAs. Early-type MgO expansive agents (MEA-71 and MEA-101) and late-type MgO expansive agents (MEA-375 and MEA-1250) were blended with each other and added into cement pastes. A total of four series of cement pastes were prepared. Figure 11a shows the expansion curves of the pastes cured in water at 40 °C, in which MEA-71 and MEA-375 were selected for blending. The paste with MEA-71 expanded rapidly, but the expansion stabilized after 28 days. The paste with MEA-375 showed less expansion in the early age, and the expansion rapidly increased after about 42 days. The pastes with blended MEAs generated more expansion than the paste with MEA-71 before 42 days, but less than the paste with MEA-375, and the expansion was between the paste with MEA-71 and the paste with MEA-375. When the amount of MEA-71 increased in the blended MEA, greater expansion was produced before 42 days. The paste with 60% MEA-71 + 40% MEA-375 generated the greatest expansion during this period. After 42 days, since the compensation effect of MEA-71 was not obvious, MEA-375 mainly played a role in expansion in this period. So, the paste with 40% MEA-71 + 60% MEA-375 generated the largest expansion after 42 days.

When MEA-1250 was chosen as the late-type MgO expansive agent, and compared with MEA-375, the paste with MEA-1250 produced more expansion in the late age (Figure 11b). So, the pastes with blended MEAs that were mixed with MEA-71 and MEA-1250 both generated more expansion in the late age than the pastes with blended MEAs that were mixed with MEA-71 and MEA-375. The time of expansion development was longer, and a clear growth trend was observed after 210 days.

Compared with compacted MEA-71, the expansion stress of compacted MEA-101 developed relatively slowly for the first few days, but then increased and developed for longer. MEA-101 was chosen as the early-type MgO expansive agent in Figure 11c,d. The difference between pastes with blended MEAs in Figure 11a and pastes with blended MEAs in Figure 11c is the type of early-type expansive agent. Before 14 days, the expansion of pastes with MEA-71 was greater than pastes with MEA-101, so the pastes with blended MEAs, in which the early-type MgO expansive is MEA-71, were bigger than those with blended MEAs using the early-type MgO expansive agent MEA-101. After 14 days, the expansion of the paste with MEA-101 exceeded that of the paste with MEA-71, so the pastes with blended MEAs, in which the early-type MgO expansive agent was MEA-101, were bigger than those with blended MEA that incorporated MEA-71 as the early-type MgO expansive agent.

Using the expansion stress development of compacted MEAs was effective for selecting the appropriate type of MEAs and the blending ratio to apply in cement pastes (Figure 11). The expansion of the cement pastes with blended MEAs lasted from the early age to late age. Their expansion was larger than the paste just with a late-type MgO expansive agent at the early age, and did not suffer from the defect where the expansion of the paste just with an early-type MgO expansive agent is not obvious in the late age. When a larger amount of early expansion was needed, the relatively low-activity MEA was a good choice for the blend. For late-type MgO expansive agent with the lower activity, the expansion of paste was greater in the late age. So, if larger late expansion is required, the lower-activity MEA can be selected for the blend. Adjustment of the blending ratio can also influence the expansion characteristics of cementitious materials, and a larger amount of the early-type MgO expansive agent in blended MEAs would produce more expansion, but the late expansion would be small. The amount of late-type MgO expansive agent can be increased when larger and later expansion is needed.

## 4. Conclusions

The expansion stress development differed among MEAs with different activities. Compacted MEA-71 and compacted MEA-101 produced expansion stress quickly at an early age, but no obvious expansion occurred at a late age, and they were classified as early-type MgO expansive agents. The time of expansion stress development of compacted MEA-71 was shorter than that of compacted MEA-101 (18 vs. 34 days, respectively), due to its higher activity, and the resulting expansion stress was smaller than MEA-101 (22.41 vs. 32.73 MPa, respectively). MEA-375 and MEA-1250 were classified as late-type MgO expansive agent, and their expansion stresses developed faster after 28 and 40 days, respectively, and finally reached 134.03 and 198.74 MPa, respectively. The expansion stress of compacted MEA-1250 developed for longer than compacted MEA-375 (190 vs. 147 days, respectively).

Measuring the expansion stress of compacted MEAs can be a novel method used to characterize the expansion property of the MEA, as it clearly demonstrates the expansion property of the MEA at different ages.

When the expansion stress of compacted MEAs stabilized, the hydration degree of periclase in the compacted MEA was about 70% for both early-type MgO expansive agents and late-type MgO expansive agents, and all compacted MEAs demonstrated a similar dense structure. The hydration of periclase in the compacted early-type MgO expansive agents was faster than in the compacted late-type MgO expansive agents, and the morphology of the formed brucite was also different.

The expansion stresses generated by blended MEAs that were mixed using MEA-71 and MEA-375 were sustained from an early age to a late age. When the amount of MEA-71 increased, the expansion stress intensified at an early age, but after 34 days, the expansion stress decreased. Additionally, the resulting expansion stress and the time of stress development also reduced. So, the expansion stress of the MEA can be adjusted as needed.

After the blended MEAs were added to the cement pastes, the expansion continued from an early age to a late age. The expansion of pastes can be regulated by adjusting the blending ratio of the two types of MEAs and selecting the activity of the two types of MEAs. Therefore, the expansion characteristics of cementitious materials with blended MEAs can be feasibly regulated.

## Figures and Tables

**Table 1:** Chemical compositions of MgO-type expansive agents (MEAs), magnesite and cement.

Sample	Chemical Composition (wt %)						
	MgO	SiO_2_	Fe_2_O_3_	Al_2_O_3_	CaO	SO_3_	Loss
MEA-71	88.72	3.80	0.79	0.60	3.83	-	1.16
MEA-101	89.58	3.78	0.81	0.61	4.01	-	0.69
MEA-375	90.88	3.47	0.61	0.40	4.10	-	0.47
MEA-1250	91.46	3.38	0.58	0.45	3.84	-	0.24
Magnesite	45.80	0.79	0.27	0.48	1.21	-	50.47
Cement	0.89	19.41	2.97	4.39	64.73	2.59	2.40

**Table 2:** Physicochemical properties of MEAs.

Sample	Periclase Content (wt %)	Crystal Grain Size (nm)	Specific Surface Area (m^2^/g)	Activity (s)
MEA-71	87.19	31.2	41.03	71
MEA-101	88.69	39.6	21.85	101
MEA-375	89.32	88.1	11.8	375
MEA-1250	90.77	>100	1.91	1250

**Table 3:** Blending ratios of the blended MEAs.

Sample	Early-Type MgO Expansive Agent (wt %)		Late-Type MgO Expansive Agent (wt %)	
	MEA-71	MEA-101	MEA-375	MEA-1250
1-1	60	-	40	-
1-2	50	-	50	-
1-3	40	-	60	-
2-1	60	-	-	40
2-2	50	-	-	50
2-3	40	-	-	60
3-1	-	60	40	-
3-2	-	50	50	-
3-3	-	40	60	-
4-1	-	60	-	40
4-2	-	50	-	50
4-3	-	40	-	60
